# Open-Air Schools

**Published:** 1920-11-27

**Authors:** 


### THE PUBLIC WELL-BEING.

### Open-Air Schools.

### AN AUTHORITATIVE APPEAL.

The advocacy of open-air education for children, which has been repeatedly put forward in these columns, is supported with remarkable emphasis in the voluminous report of the Chief Medical-Officer of the Board of Education, to which reference was made last week. The development of provision of facilities for open-air education, notwithstanding the supreme importance of this matter, is still very limited, and the chief medical officer complains that a number—he might have added a large number—of local education authorities are slow in appreciating the value of such provision. It has been demonstrated undeniably for some years past that the response of the children, as regards their physical and mental content, to open-air conditions of school life is invariable. In view of the certainty of the benefit there to be derived, authorities are being strongly urged (1) To instruct their school medical officer to report to them both as to the particular need of their areas for open-air schools or classes, and as to the closer study of the effect of the open-air system upon different types of debilitated children; (2) To secure opportunities to institute or extend arrangements for providing education under open-air conditions, *e.g.*, the institution of playground classes, the establishment of schools or classrooms in wooden structures, such as the huts used in connection with the war. A large number of these buildings will be available and can easily be converted on open-air lines.

The vigorous views expressed by the Chief Medical Officer concerning the general question are in such close accord with those which we have ourselves expressed, and are so clear and precise, that they deserve quotation in full. "Fresh air and sunlight," he says, "exercise, adequate nutrition, and sufficient rest are the finest life-giving and remedial agents that we possess, and yet we consign the vast majority of school children for five or six hours in five days a week to buildings, in many cases ill-ventilated and where sunlight has little direct access, and to a school system in which exercise, nutrition, and rest play only a very subsidiary part.

"It is not, I think, much wonder that the children are often listless and inattentive, anæmic and ailing, and that they do not grow mentally and physically as rapidly and completely as they undoubtedly should. Why is it that in the interests of the young child the simplest facilities, often *available and at hand for adoption*, are so neglected? It cannot be because of uncertainty as to their effect, for the numerous experiments as regards open-air education, which have been conducted during the past ten or fifteen years, have abundantly proved its worth. In some cases the inaction would appear to be due to a want of consideration and enterprise on the part of those primarily responsible for the organisation of the school; in others to an unwillingness to appreciate and profit by experience, however incontestable and unwavering.

"The demand for the provision of open-air facilities for education in all areas is insistent, and it should, I think, be met in greater degree than has been the case hitherto. Local education authorities will find that in return they will secure a much higher standard of health throughout the schools, which will be reflected in an increased attendance, and substantial improvement in the health and virility of the children."

These remarks are at once a serious indictment and a powerful appeal. Next week we hope to give some account of the system of holiday camps now in operation in many parts of the country, particularly with reference to London, a subject on which Dr. Hamer, the County School Medical Officer for London, speaks with authority.

